# Diagnostic accuracy of two commercial SARS-CoV-2 antigen-detecting rapid tests at the point of care in community-based testing centers

**DOI:** 10.1371/journal.pone.0248921

**Published:** 2021-03-31

**Authors:** Alice Berger, Marie Therese Ngo Nsoga, Francisco Javier Perez-Rodriguez, Yasmine Abi Aad, Pascale Sattonnet-Roche, Angèle Gayet-Ageron, Cyril Jaksic, Giulia Torriani, Erik Boehm, Ilona Kronig, Jilian A. Sacks, Margaretha de Vos, Frédérique Jacquerioz Bausch, François Chappuis, Adriana Renzoni, Laurent Kaiser, Manuel Schibler, Isabella Eckerle

**Affiliations:** 1 Division of Infectious Disease, Geneva University Hospitals, Geneva, Switzerland; 2 Geneva Centre for Emerging Viral Diseases, Geneva University Hospitals, Geneva, Switzerland; 3 CRC & Division of Clinical-Epidemiology, Department of Health and Community Medicine, University of Geneva & University Hospitals of Geneva, Geneva, Switzerland; 4 Department of Microbiology and Molecular Medicine, University of Geneva, Geneva, Switzerland; 5 Foundation for Innovative New Diagnostics, Geneva, Switzerland; 6 Department of Primary Care, Geneva University Hospitals, Geneva, Switzerland; 7 Division of Laboratory Medicine, Laboratory of Virology, Geneva University Hospitals, Geneva, Switzerland; University of Cape Town Faculty of Health Sciences, SOUTH AFRICA

## Abstract

**Objectives:**

Determine the diagnostic accuracy of two antigen-detecting rapid diagnostic tests (Ag-RDT) for SARS-CoV-2 at the point of care and define individuals’ characteristics providing best performance.

**Methods:**

We performed a prospective, single-center, point of care validation of two Ag-RDT in comparison to RT-PCR on nasopharyngeal swabs.

**Results:**

Between October 9^th^ and 23^rd^, 2020, 1064 participants were enrolled. The Panbio^TM^ Covid-19 Ag Rapid Test device (Abbott) was validated in 535 participants, with 106 positive Ag-RDT results out of 124 positive RT-PCR individuals, yielding a sensitivity of 85.5% (95% CI: 78.0–91.2). Specificity was 100.0% (95% CI: 99.1–100) in 411 RT-PCR negative individuals. The Standard Q Ag-RDT (SD Biosensor, Roche) was validated in 529 participants, with 170 positive Ag-RDT results out of 191 positive RT-PCR individuals, yielding a sensitivity of 89.0% (95%CI: 83.7–93.1). One false positive result was obtained in 338 RT-PCR negative individuals, yielding a specificity of 99.7% (95%CI: 98.4–100). For individuals presenting with fever 1–5 days post symptom onset, combined Ag-RDT sensitivity was above 95%. Lower sensitivity of 88.2% was seen on the same day of symptom development (day 0).

**Conclusions:**

We provide an independent validation of two widely available commercial Ag-RDTs, both meeting WHO criteria of ≥80% sensitivity and ≥97% specificity. Although less sensitive than RT-PCR, these assays could be beneficial due to their rapid results, ease of use, and independence from existing laboratory structures. Testing criteria focusing on patients with typical symptoms in their early symptomatic period onset could further increase diagnostic value.

## Introduction

The Coronavirus Disease 19 (COVID-19) pandemic, caused by SARS-CoV-2, has led to an unprecedented public health crisis. Diagnostic strategies that are low cost, rapid, and easily accessible are critical to control the pandemic. While RT-PCR based assays remain the standard for the detection of emerging respiratory viruses [1, 2], the need for high through-put virus detection has fueled development of Antigen-detecting rapid diagnostic tests (Ag-RDT), that can be performed at the point of care (POC) [3].

Such laboratory-independent tests could allow for control of the pandemic by quickly isolating individuals during their contagious period to prevent further transmission. These tests can affordably help overcome overwhelmed diagnostic laboratories and global PCR-reagent shortages [4, 5]. Given that viral load, as measured by RNA copies, peaks near symptom onset [6–8] and contagiousness begins even earlier [9, 10], RDTs may have the highest sensitivity (SN) in the most contagious individuals. Reported SNs of Ag-RDTs vary widely, and manufacturer reported SNs are often substantially higher than those of independent assessments [11]. Previous studies with NPS rapid antigenic tests (Panbio) for detection of SARS-CoV-2 shows the sensitivity of 95% for Ct ≤25, and 85% for Ct <30 in symptomatic patients and the sensitivity near of 80% for a period of less than 7 days from symptom onset but the overall sensitivity in this population seems low at 67.3% [12]. A study on two NPS RDT (Panbio and SD Biosensor) versus PCR in symptomatic population found an overall sensitivity of 73.8% and 68.5%; for a CT value< 30, sensitivities of two RDT are respectively 87% and 81.4% in a population with mostly symptomatic patients [13]. Lambert-Niclot et al. found a sensitivity of 50.0% with RDT NPS (Respi-Strip) versus PCR and for the CT value <25CT, sensitivity was 82.2% [14].

The World Health Organization’s (WHO) Ag-RDT target product profile aims at SNs ≥80% and specificities (SP) of ≥97% [5, 15]. Thus, we sought to evaluate the performance of two commercially available Ag-RDTs through a prospective, single-center POC validation in comparison to RT-PCR for detecting SARS-CoV-2 using nasopharyngeal swabs (NPS). We evaluate also the performance of these tests according to the duration of symptoms and viral loads of patients mostly symptomatic.

## Methods

### Ethics

The study was approved by the Cantonal ethics committee (Nr. 2020–02323). All study participants and/or their legal guardians provided written informed consent.

### Setting

The study was performed in two geographically different testing centres run by our institution, the Geneva University Hospital. Both centres are supervised by the same team, and did not differ in their infrastructure, so we analyzed them as a single centre study.

### Study design and participants

The primary objective of this prospective study was to assess the diagnostic accuracy (SN and SP) of the Ag-RDTs compared to the reference RT-PCR. Participants were ≥16 years old, with suspected SARS-CoV-2 infection according to the local governmental testing criteria. This included suggestive symptoms for COVID-19 and/or recent exposure to a SARS-CoV-2 positive person. Asymptomatic individuals were included if they were notified by the Swiss COVID-19 app or by local health authorities after contact to a confirmed case.

### Study procedures

For each participant, two NPS were collected. The first was a standard flocked swab placed in viral transport media (VTM), used routinely for viral genome detection by RT-PCR. The second NPS, provided in the Ag-RDT kit, was obtained from the contralateral nostril and was performed as recommended by the manufacturer. Both swabs were taken by the same trained nurse. All Ag-RDTs were performed immediately at the sample collection site. Adequate personal protective equipment was used while collecting the NPSs and performing the RDTs.

### Data collection

Clinical data were collected for each patient upon presentation with a questionnaire including the number of days post symptom onset (DPOS), known contact to a previous SARS-CoV-2 infected person, comorbidities and type of symptoms. The following symptoms were recorded: rhinorrhea (runny nose), odynophagia, myalgia, chills, dry cough, productive cough, red expectoration, fever (anamnestic), anosmia/ageusia (loss of smell or taste), gastrointestinal symptoms, asthenia, dyspnea, thoracic pain and headache.

Comorbidities included in the questionnaire were hypertension, cardiovascular disease, chronic pulmonary disease, diabetes, chronic renal failure, active cancer including lymphoproliferative disease, severe immunosuppression, immunosuppressive therapy, pregnancy, and obesity (BMI >40 kg/m^2^).

### Ag-RDT testing

The two validated Ag-RDTs were Panbio^TM^ COVID-19 Ag Rapid Test Device (Abbott Rapid Diagnostics, US) and Standard^TM^ Q COVID-19 Ag Test (SD Biosensor, distributed by Roche, Switzerland). Both Ag-RDTs were used as recommended by the manufacturers, using only materials provided in the kit. Both assays were manually read, with two individuals reading the results separately. In case of discordant results, the two validators sought a consensus. All visible bands were considered a positive result. All Ag-RDT results were photographically documented.

### RT-PCR testing

All participants were tested by a single well, dual target RT-PCR assay for SARS-CoV-2 (cobas® SARS-CoV-2 Test, Cobas 6800, Roche, Switzerland) using NPS in 3mL VTM. For further analysis, only cycle threshold (Ct) values for the E-gene were used. For calculation of viral loads (VL) as SARS-CoV-2 genome copy numbers per mL, a standard curve was obtained by using a quantified supernatant from a cell culture isolate of SARS-CoV-2. All VLs were calculated from the Ct-values, according to log10 SARS-CoV-2 RNA copies/ml = (Ct-44.5)/-3.3372 for Cobas [16, 17].

### Statistics

Over a 2-week period, we enrolled all patients who met the SARS-CoV-2 testing criteria; 535 and 529 patients were enrolled during the first and second week, respectively. The target sample size was 530, as it would have sufficient power to generate a 95% confidence interval (CI) with a lower bound above the WHO target of 80%, if the prevalence was 25% and the measured SP was ≥87.5%.

All continuous variables were presented by their mean ±standard deviation (SD) and median (interquartile range, IQR), categorical variables were presented by their frequencies and relative proportions. For comparisons of continuous variables, we used a nonparametric Mann-Whitney test due to small sizes; for comparisons of categorical variables, we either performed Chi^2^ or Fischer’s exact tests, depending on applicability.

To enable Ag-RDT result combination, we performed a Bayesian t-test on their sensitivities and specificities. To be able to conduct the t-test, the confidence intervals of both sensitivities and specificities were converted into standard deviation to allow for the t-test to be conducted. The test computes a Bayesian Factor (BF) that allows comparison of the probability of observing our data under H_0_ (both tests are equal in term of SN and SP) and H_1_ (both tests are different). All analyses were performed using STATA version intercooled 16 (Stata Corp., College Station, TX, USA). Statistical significance was defined as p<0.05 (two-sided).

## Results

Between October 9^th^ and October 23^rd^, 2020, 1064 participants were enrolled and included in the analysis. 535 participants were tested with the Panbio Ag-RDT from October 09^th^ to 16^th^ and 529 participants were tested with the Standard Q Ag-RDT from October 19^th^ to 23^rd^, 2020.

Characteristics of the study population are shown in **Table 1**. The mean age of the study participants was 34.9 years (SD ±10.9) with 53.8% being female. The mean DPOS to testing was 2.7 (SD ±1day). Overall, 29.6% of participants were positive by RT-PCR with a mean Ct-value of 22.5 (SD ±5.1), corresponding to a VL of 1.8E7 SARS-CoV-2 copies/mL. Most patients (97.8%) were symptomatic upon presentation at the testing centre, with only 3 reporting no symptoms. Symptoms information was missing for 4 patients. The study population tested with the Standard Q was younger than that tested with the Panbio assay (34.9 ±10.9 vs 38.5 ±13.6 years, respectively, p<0.001) and DPOS differed slightly (2.9±1.5 vs. 2.6±2.0 days, respectively, p = 0.0125). Ct-values did not differ significantly between the two groups (p = 0.450): 22.6 (SD ±4.9) in the Standard Q group vs. 22.4 (SD ±5.4) in the Panbio group, corresponding to 1.7E7 and 1.9E7 SARS-CoV-2 RNA copies/mL, respectively. The RT-PCR positivity rate was 23.2% and 36.1% for the population tested with the Panbio and the Standard Q, respectively, corresponding to an increase in the overall PCR positivity rate and reflecting the rapidly increasing local incidence during the time of this study.

**Table 1:** Characteristics of the study population.

Characteristics	Standard Q	Panbio	Combined	p-value
	(n = 529)	(n = 535)	(n = 1064)	
Mean age (±SD, median)	34.9 (±10.9, 33)	38.5 (±13.6, 36)	36.7 (±12.5, 34)	<0.001
Sex distribution, n (%)				0.966
Women	285 (53.9)	287 (53.6)	572 (53.8)	
Men	244 (46.1)	248 (46.4)	492 (46.2)	
Mean DPOS to RT-PCR (±SD, median)	2.9 (±1.5, 3)	2.6 (±2.0, 2)	2.7 (±1.9, 2)	0.0125
Result of RT-PCR, n (%)				<0.001
Negative	338 (63.9)	411 (76.8)	749 (70.4)	
Positive	191 (36.1)	124 (23.2)	315 (29.6)	
Mean Ct (±SD, median) (n = 315)	22.6 (±4.9, 21.8)	22.4 (±5.4, 21.0)	22.5 (±5.1, 21.5)	0.450

SD, standard deviation; RT-PCR, reverse transcription polymerase chain reaction; DPOS, days post symptom onset; Ct, cycle threshold.

The overall test performance for the Standard Q was 89.0% SN (95%CI: 83.7–93.1) and 99.7% SP (95%CI: 98.4–100%). The overall test performance for the Panbio assay was 85.5% SN (95%CI: 78.0–91.2) and 100% SP (95%CI: 99.1–100) (**Table 2**). The absolute numbers of positive and negative detections per method (by RT-PCR and Ag-RDT) and number of true positive, false negatives, false positives and true negatives can be found in **Table 3.**

**Table 2:** Overall SN, SP, positive and negative predictive value of Standard Q and Panbio SARS-CoV-2 Ag-RDT.

Characteristics	Standard Q	Panbio	Combined
SN, % (95%CI)	89.0 (83.7–93.1)	85.5 (78.0–91.2)	87.6 (83.5–91.0)
SP, % (95%CI)	99.7 (98.4–100)	100 (99.1–100)	99.9 (99.3–100)
Positive predictive value, % (95%CI)	99.4 (96.8–100)	100 (96.6–100)	99.6 (98.0–100)
Negative predictive value, % (95%CI)	94.1 (91.2–96.3)	95.8 (93.4–97.5)	95 (93.3–96.5)

Positivity rate at the time of study for Standard Q was 36.1% and at the time of study for Panbio was 23.2%.

**Table 3:** RT-PCR and Ag-RDT results.

	Total	Ag-RDT +/ RT-PCR + (TP)	Ag-RDT—/RT-PCR + (FN)	Ag-RDT +/ Rt-PCR—(FP)	Ag-RDT -/RT-PCR—(TN)
Standard Q	529	170	21	1	337
Panbio	535	106	18	0	411

+, positive; -, negative; TP, true positive; FN, false negative; FP, false positive.

**Fig 1:**
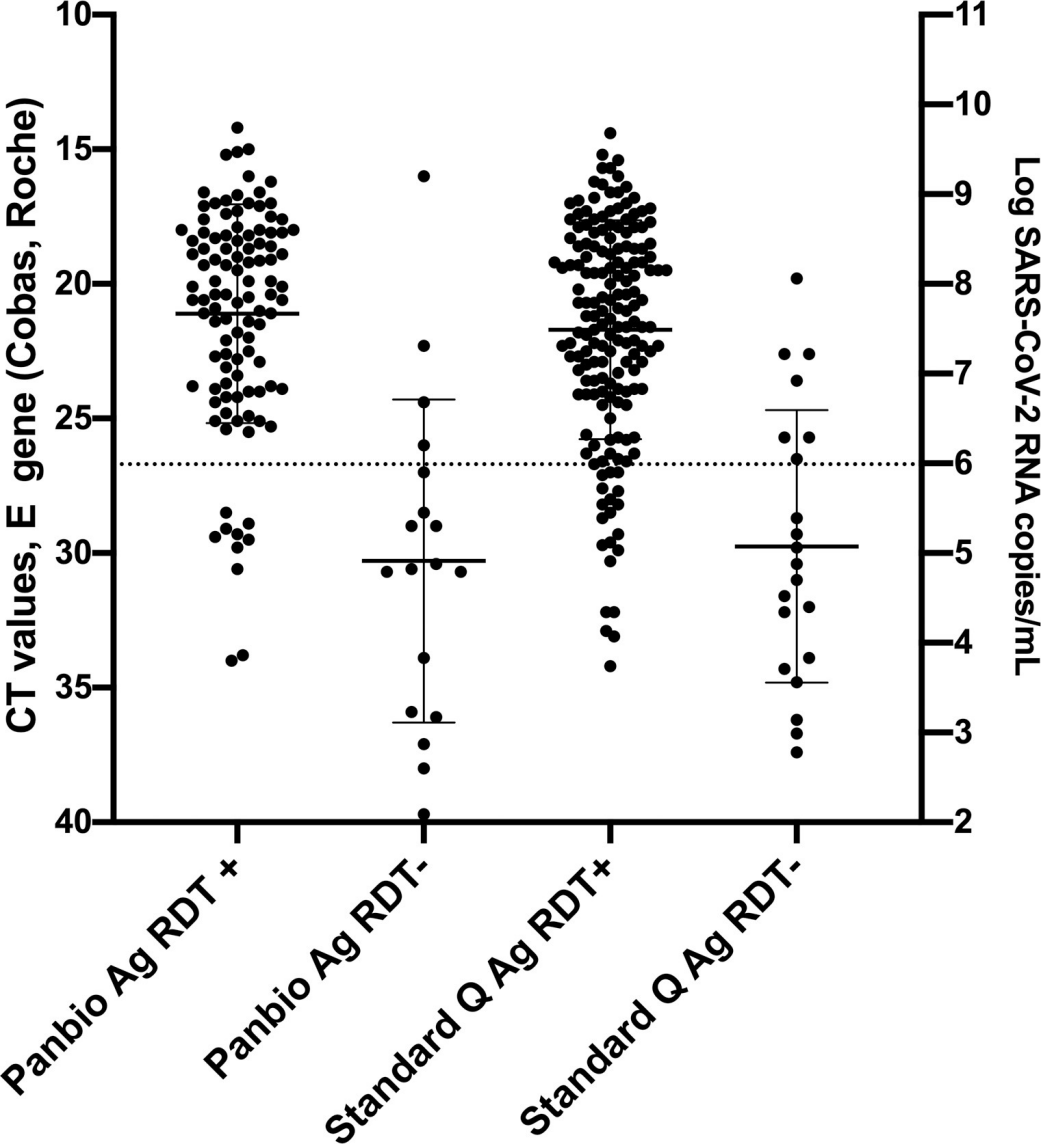
Ct values, viral load and Ag-RDT results for RT-PCR-positive individuals tested with Standard Q (n = 191) and Panbio (n = 124). Horizontal bars represent median and standard deviation. Dotted line: Ct value of 26.7 or 1E6 SARS-CoV-2 RNA copy numbers/mL.

**Fig 2:**
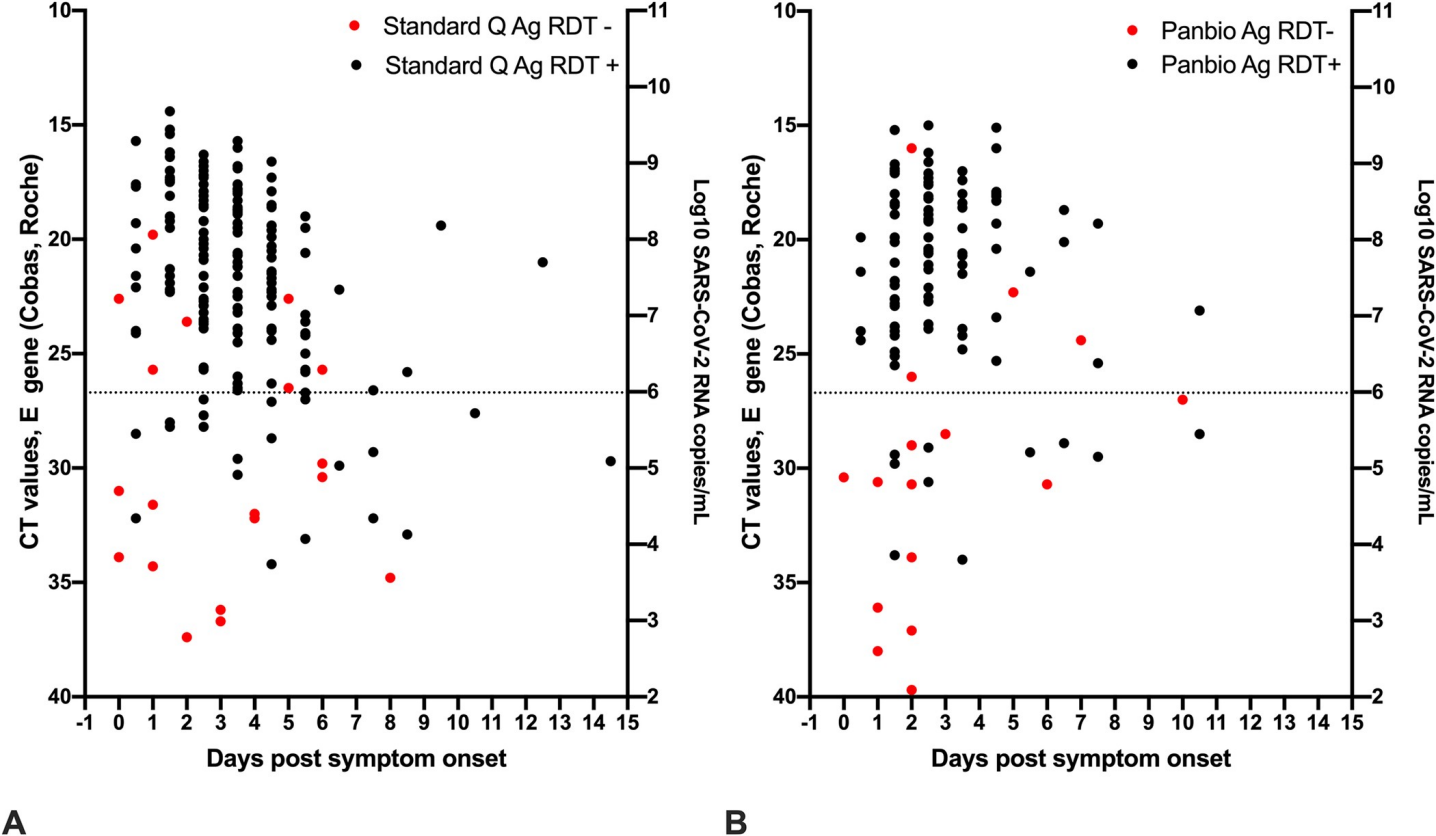
Ct values, viral load, days post symptom onset and Ag-RDT results for 190 patients tested with Standard Q (**A**) and 116 patients tested with Panbio (**B**) for which information on day of symptom onset was available. Dotted line: Ct value of 26.7 or 1E6 SARS-CoV-2 RNA copy numbers/mL.

Ct-values of samples with positive Ag-RDT results ranged from 14.2–34.0 and 14.4–34.2 for Panbio and Standard Q (p = 0.1766), respectively, while Ct-values of samples of samples that tested falsely negative by Ag-RDT ranged from 16.0–39.7 and 19.8–37.4 (p = 0.7998), respectively. Median Ct-values of Ag-RDT positive samples (Panbio: 20.4, IQR: 18.1–23.8; Standard Q: 21.2, IQR 18.6–24) were lower than those of Ag-RDT negative samples (Panbio: 30.5, IQR: 27–35.9; Standard Q: 30.4, IQR: 25.7–33.9) (**Fig 1**). Furthermore, we evaluated overall Ag-RDT results in relation to Ct-values/viral load as well DPOS (**Fig 2**). False-negative results occurred in both assay across all DPOS.

We compared SN and SP between the two Ag-RDTs and concluded that we could accept, with high probabilities, (respectively likelihood ratio of BF_01_ = 10.2 and 11.9) the hypothesis of equivalent SN and SP. Based on this, a combined SN of 87.6% (95%CI: 83.5–91.0) and a combined SP of 99.9% (95%CI: 99.3–100) for both Ag-RDTs were calculated with a positive predictive value of 99.6% (95%CI: 98.0–100) and a negative predictive value of 95.0% (95%CI: 93.3–96.5). In order to identify subpopulations in which maximal SN could be reached with these tests, we analyzed SN by DPOS, Ct-values as determined by RT-PCR, type of symptoms, comorbidities, and previous contact with a confirmed SARS-CoV-2 infection.

**Fig 3:**
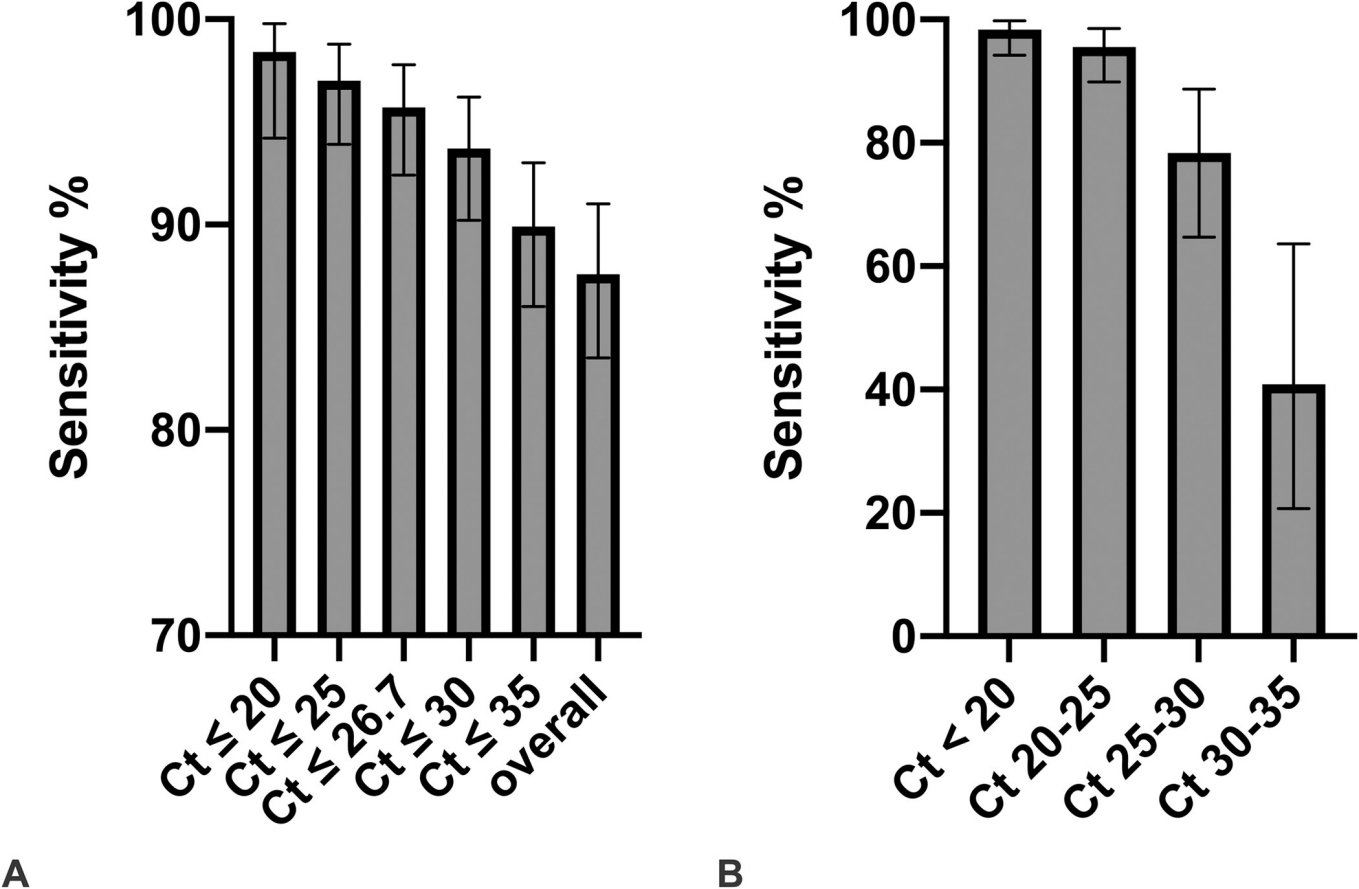
**A.** Combined SN of the two Ag-RDTs according to Ct-values of the RT-PCR. **B.** Combined SN of the two Ag-RDTs according to subgroups of Ct-values of the RT-PCR. Ct values correspond to the following SARS-CoV-2 RNA copy numbers/mL: Ct 20: 1.0E8; Ct 25: 3.2E6; Ct 26.7: 1E6; Ct 30: 1.0E5, Ct 35: 3.2E3.

Combined SN varied according to Ct-values: it was highest in samples with low Ct-values, with a SN of 98.4% (95% CI: 94.2–99.8) for Ct ≤20 (≤1.0E8 SARS-CoV-2 copies/mL), decreased slightly to 95.5% (95%CI: 89.9–98.5) for 20 < Ct ≤25 (<1.0E8 SARS-CoV-2 copies/mL ≤ 3.2E6), dropped further to 89.9% (95%CI: 86.0–93.0) for Ct ≤35 (≤3.2 E3 SARS-CoV-2 copies/mL) and was lowest (only 40.9% (95%CI: 20.7–63.6)) for 30< Ct ≤35 (<1.0E5 SARS-CoV-2 copies/mL ≤3E3) (**Fig 3**). The SN for all samples with a Ct value ≤26.7 (≤1E6 SARS-CoV-2 copies/mL), an assumed cut-off for presence of infectious virus, was 95.7% (95%CI: 92.4–97.8).

**Fig 4:**
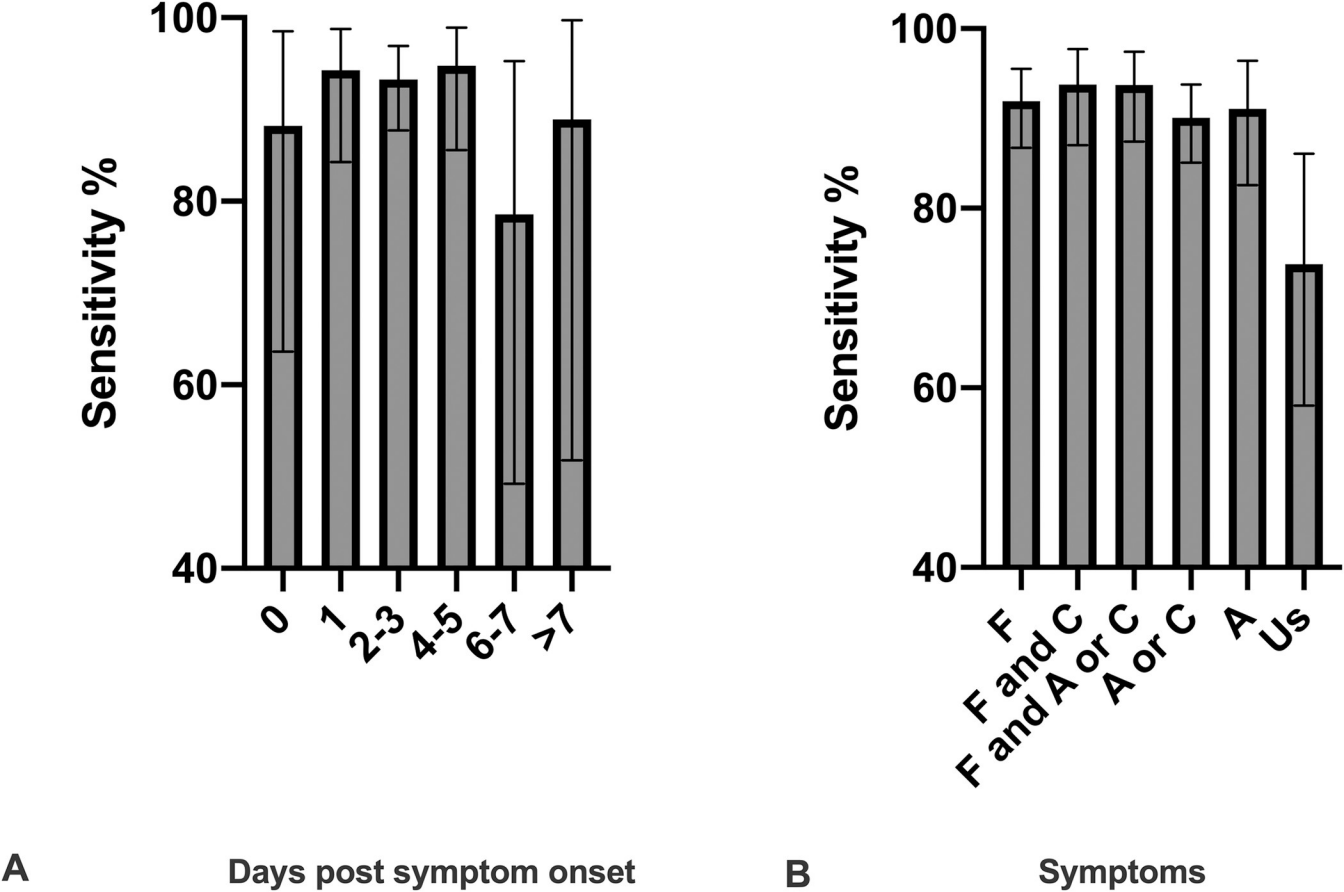
**A.** Combined SN of the two Ag-RDTs according to days post symptom onset. Number of patients per category: Day 0, n = 17; day 1, n = 53, day 2–3, n = 135; day 4–5, n = 58; day 6–7, n = 14; > 7 days, n = 9). **B.** Combined SN of the two Ag-RDTs according to symptoms F, fever/chills; C, cough, A, anosmia/ageusia (loss of smell or taste), Us, unspecific symptoms (all other symptoms excluding fever/chills, cough and anosmia/angeusia). Number of patients per category: F, n = 172; F and C, n = 97; F and A or C, n = 111; A or C, n = 202; A, n = 79; Us, n = 42.

SN increased with DPOS, from 88.2% at 0 DPOS (95%CI: 63.6–98.5) to 94.3% (95%CI: 84.3–98.8, p = 0.030) at 1 DPOS, and remained high until 5 DPOS. The highest SN was seen between 1 DPOS and 4–5 DPOS, ranging from 94.3% (95%CI: 84.3–98.8) to 94.8% (95%CI: 85.6–98.9), with a decline after 5 DPOS (**Fig 4A**).

Additionally, we analyzed SN according to specific symptoms, differentiating between typical COVID-19 symptoms (fever/chills, cough and anosmia/ageusia) and more non-specific symptoms of respiratory infection (all other symptoms reported). The highest SN of 93.8% (87.0–97.7) was observed for patients presenting with fever/chills and cough at the time of testing, followed by patients presenting with anosmia/ageusia or cough and fever/chills with a SN of 93.7% (95%CI: 87.4–97.4), but only 73.8% (95%CI: 58.0–86.1) in patients presenting with non-specific signs (**Fig 4B**). No difference in SN was seen between patients with (89.3%, 95%CI: 71.8–97.7, n = 28) or without (87.5%, 95%CI: 83.1–91.1, n = 287) comorbidities (p = 0.999). Typical symptoms were more frequent in patients with comorbidities (100%, 15/15 patients) than in patients without comorbidities (86.5%, 96/138 patients) (p = 0.012), however sample size was small. No difference was seen in patients with or without contact with a recently positive case (p = 0.065). We further analysed by DPOS, and found that the highest SN was seen in patients with fever/chills and presenting between 1 and 5 DPOS, at 95.7% SN (95%CI: 91.0–98.4).

## Discussion

This study provides an independent, POC validation of two commercial Ag-RDTs relative to RT-PCR and according to demographic and clinical information. This combined validation of two similar assays provides performance data in a real-life high incidence test setting.

Both RDTs performed well with an overall SN of 87.6% (95%CI: 83.5–91) and a very high SP of 99.9% (95%CI: 99.3–100) during a time of very high SARS-CoV-2 weekly incidence (375/100,000 to 824/100,000 inhabitants) and a SARS-CoV-2 RT-PCR positivity rate >20%. SN was higher in sub-populations with earlier DPOS numbers and characteristic COVID-19 symptoms. Importantly at 0 DPOS, the SN is lower than during the subsequent days, and the sensitivity drops rapidly when the Ct-values increase above a threshold of 30, mostly after 6–7 days.

The highest VL and thus transmission probability occurs within the first week of symptom onset, with VLs peaking around the time of symptom onset [6]. Culturable virus has been predominantly found in the first week after symptom onset, down to a VL around 1E6 copies/ml [17–20], which has been set as a detection cut-off for Ag-RDTs by the WHO [15]. The SN of the Ag-RDTs validated here, for VLs compatible with contagiousness, was 95.7%. Correspondingly, the highest Ag-RDT SN was also observed at early DPOS numbers and in patients with low Ct-values, again suggesting reliable identification of contagious individuals.

Our findings at the POC are in line with other validations performed in different countries and prevalences, although study designs and specimens used varied considerably between studies. Standard Q was reported to have SNs between 70.6–88.7%, while SP remained high throughout these studies between 97.6–100% [21–27]. A clinical study performed similarly to ours in a much lower-incidence setting (<1% RT-PCR positivity rate), found a SN/SP of 76.6%/100%, using a mixture of NPS and combined oro- and naso-pharyngeal swabs from a total of 2417 participants with 47 RT-PCR positive samples yielding 36 Ag-RDT [21].

For Panbio, other studies have reported SNs ranging from 73.3–91.7% with SP in the range of 94.9–100% [28–31]. Notably, the highest reported SN of 91.7%/98.9% comes from a study, not done at a POC, using frozen NPS specimens [28]. Although the use of frozen samples is possible for RDTs [22, 28], it is not their intended use and does not represent their use in the field. It is also unknown if a freeze-thaw cycle can affect the accessibility of viral antigens.

While significant variation in Ag-RDT SN is observed across studies, there is remarkable similarity within certain Ct-value ranges–although caution must be exercised comparing Ct-values of different assays. For Ct-values of <25, the Standard Q test was reported to have a SN of 100% [21], while the Panbio was reported to have a SN of 97.1% [30] or 98.2% [28], which is in agreement with our results of 97%. In contrast to most other validations, we did observe false-negative Ag-RDT results in patients with high VLs across a range of DPOS. These patients are likely contagious and able to transmit SARS-CoV-2, with VLs associated with culturable virus.

We did not find any validation of Ag-RDTs that has analyzed SN based on type of symptoms, which could be an additional factor for testing algorithms. This finding also highlights that Ag-RDTs are a valid diagnostic tool in symptomatic individuals, while the benefit of their use in asymptomatic individuals or in patients with atypical or minor symptoms remains to be investigated. In a situation with sufficient RT-PCR capacity, testing algorithms focusing on a subgroup of symptomatic patients, while testing others with more sensitive RT-PCR might be an option to make the best use of Ag-RDTs. In our data set, the best SN is found in symptomatic individuals with symptoms suggestive of COVID-19, between 1 and 5 DPOS.

Our study has several strengths. The Ag-RDTs were performed at the POC in parallel to RT-PCR, and is one of the largest in terms of RT-PCR positive individuals. The test population represents a population screened for public health intervention, and not for diagnostic purposes in a hospital setting: mainly young unhospitalized adults without comorbidities, who mostly had typical and mild COVID-19 symptoms. This currently describes the majority of SARS-CoV-2 infected individuals, and an important group for limiting community transmission.

Although Ag-RDTs are less sensitive than RT-PCR and, we saw false-negative Ag-RDT results in patients with high VL, the public health benefit of quickly identifying a large proportion of infected individuals would still outweigh the disadvantages of occasional missed diagnoses [32].

Furthermore, our validation showed very high SP, with only one false-positive Ag-RDT result in the whole study. Interestingly, the patient with this putative false-positive tested positive 3 days later for SARS-CoV-2 by RT-PCR. This study was conducted in a high prevalence setting, thus extrapolating the findings of our study to low prevalence settings must be done with caution.

In conclusion, we show good diagnostic accuracy of both Ag-RDTs, especially for rule-in purposes of infected individuals and in patients with certain criteria. The SN for identification of SARS-CoV-2 infections, rapidity of results, and the laboratory-independence make these Ag-RDTs promising tools for SARS-CoV-2 infection control.
